# Neurosecretory Protein GL Promotes Normotopic Fat Accumulation in Male ICR Mice

**DOI:** 10.3390/ijms23126488

**Published:** 2022-06-10

**Authors:** Yuki Narimatsu, Daichi Matsuura, Eiko Iwakoshi-Ukena, Megumi Furumitsu, Kazuyoshi Ukena

**Affiliations:** Laboratory of Neurometabolism, Graduate School of Integrated Sciences for Life, Hiroshima University, Higashi-Hiroshima, Hiroshima 739-8521, Japan; d214243@hiroshima-u.ac.jp (Y.N.); matsuura.kozinyou@gmail.com (D.M.); iwakoshi@hiroshima-u.ac.jp (E.I.-U.); mfurumi@hiroshima-u.ac.jp (M.F.)

**Keywords:** neurosecretory protein GL, hypothalamus, neuropeptide, obesity, ICR strain, transgenic mice

## Abstract

Neurosecretory protein GL (NPGL) is a small secretory protein identified in the hypothalamus of birds and mammals. We recently reported that NPGL exerts obesogenic effects in obesity-prone C57BL6/J mice. However, whether NPGL elicits adiposity in different mouse strains is poorly understood. In this study, we generated transgenic mice overexpressing *Npgl* using the ICR strain (*Npgl* Tg mice) to elucidate the obesogenic effects of NPGL in different strains. *Npgl* Tg mice showed increased white adipose tissue (WAT) mass. Although the mass of brown adipose tissue (BAT) was slightly altered in *Npgl* Tg mice, hypertrophy of lipid droplets was also observed in BAT. In contrast, fat accumulation was not induced in the liver, with the upregulation of mRNAs related to hepatic lipolysis. These results support the hypothesis that NPGL causes obesity in several strains and species. This report highlights the pivotal role of NPGL in fat accumulation in adipose tissues and contributes to the elucidation of the biological mechanisms underlying obesity and metabolic diseases in heterogeneous populations.

## 1. Introduction

In the 21st century, called the “Age of Plenty” in developed countries, obesity has become a major health concern. Obesity increases the risk of severe coronavirus disease 2019 (COVID-19) and metabolic syndromes such as hyperglycemia, hyperlipidemia, and hypertension [1,2,3,4]. The hypothalamus, a key regulator of whole-body homeostasis, is well known for its involvement in feeding behavior [5]. Excessive energy intake over expenditure readily causes fat accumulation and eventually obesity. Hence, many researchers have investigated the regulation between the hypothalamus and feeding behavior in rodents. For instance, neuropeptide Y (NPY), agouti-related protein (AgRP), orexin, galanin, and melanin-concentrating hormone (MCH) potently stimulate feeding behavior [6,7,8,9]. In contrast, α-melanocyte-stimulating hormone (α-MSH), derived from proopiomelanocortin (POMC), corticotropin-releasing hormone (CRH), and cocaine- and amphetamine-regulated transcript (CART), exert anorexigenic effects in rodents [10,11,12]. In addition, peripheral hormones regulate energy homeostasis, especially in the control of fat depots. Leptin secreted from adipose tissue not only inhibits food intake but also reduces fat mass due to accelerated catabolism [13]. Conversely, insulin derived from the pancreas is one of the most well-known anabolic hormones that promotes glucose uptake and suppresses lipolysis [14]. Therefore, the central and/or peripheral endocrine system intricately controls feeding behavior and lipid metabolism, ultimately leading to obesity.

The metabolic states of experimental model mice depend on their genetic background. Although MCH-deficient mice backcrossed with C57BL/6 mice were susceptible to diet-induced obesity (DIO), those backcrossed with 129 SvJ showed a slight gain in body mass [15]. A recent study has shown that hepatic inflammation and fibrosis are influenced by the genetic background of the mice [16]. Furthermore, the trends between the change in fat mass and degree of exercise were dependent on the genotype of the mice [17]. Given that the genetic polymorphisms of mice, as well as humans [18], greatly contribute to metabolic status, further studies using various strains of mice will help to elucidate the molecular mechanisms of obesity in heterogeneous populations. However, studies focusing on the association of the endocrine system with the strain of experimental mice are limited.

We recently reported the identification of a novel cDNA encoding a small secretory protein, neurosecretory protein GL (NPGL), in the hypothalamus of birds and mammals [19,20,21]. As *Npgl* is evolutionarily conserved in vertebrates, including humans, rats, mice, and chicks, it is possible that NPGL plays important physiological roles in some species [22]. Chronic intracerebroventricular (i.c.v.) infusion of NPGL induces a significant increase in body mass gain and food intake in chicks [23]. In C57BL/6J mice, *Npgl* mRNA levels were elevated by fasting and reduced by short-term high-fat diet (HFD) feeding [20]. Furthermore, central injection of NPGL increases food intake [20]. Taken together, these results suggest that NPGL participates in energy homeostasis in chicks and mice. Moreover, our latest study using C57BL/6J mice showed that NPGL leads to lipid accumulation in white adipose tissue (WAT) in the short term and eventually obesity [24]. These results indicate that NPGL is a novel neuronal regulator of lipid metabolism in mice. However, most previous reports on NPGL have employed strain C57BL/6J, and there are few studies regarding NPGL in other mouse strains.

In this study, we generated a transgenic mouse line with ubiquitous overexpression of *Npgl* backcrossed onto the ICR outbred strain (*Npgl* Tg mouse) and analyzed its phenotype to assess whether NPGL elicits fat accumulation in a mouse strain other than C57BL/6J. Here, we show that NPGL stimulates lipid accumulation in the ICR strain and supports potent lipogenic effects in rodents.

## 2. Results

### 2.1. Production of Tg Mouse

**Figure 1:**
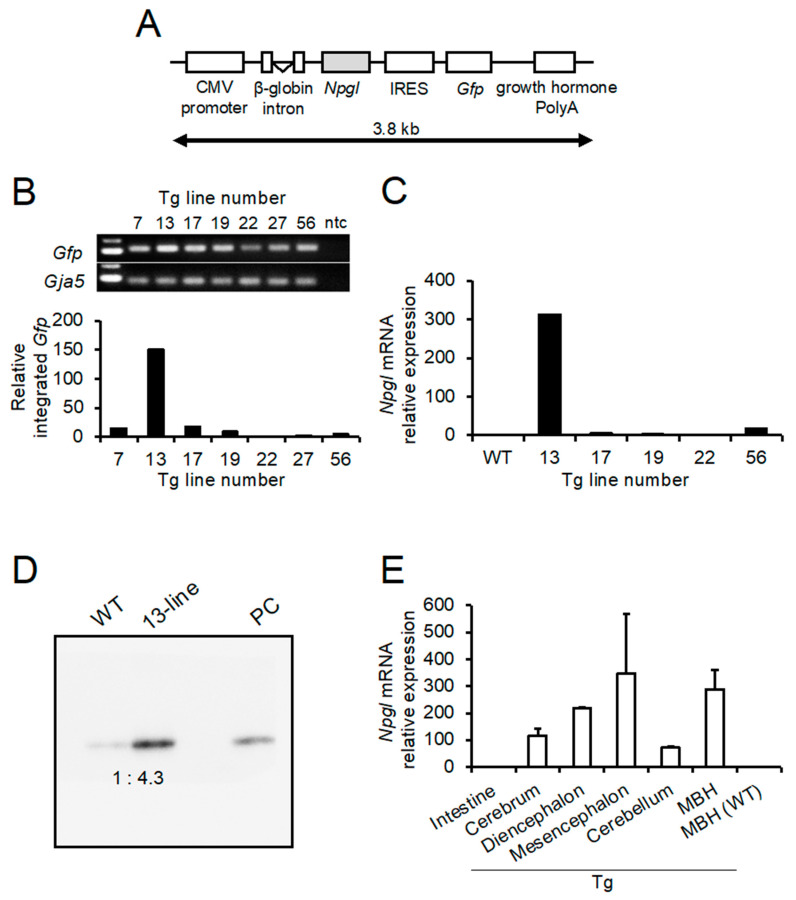
Generation of *Npgl* Tg mice and line selection using phenotypic analysis. (**A**) Structure of the 3.8 kb transgene integrated into the genomic DNA of *Npgl* Tg mice. *Npgl* is under the control of the ubiquitous CMV promoter. IRES enables co-expression of *Gfp* with *Npgl*. The β-globin intron was inserted between the CMV promoter and *Npgl*. (**B**) Genomic DNA analysis (top) and quantification of integrated *Gfp* using qRT-PCR (bottom). Genomic DNA analysis revealed that the transgene was integrated into seven F0 mice. The numbers of transgenic mice were 7, 13, 17, 19, 22, 27, and 56. *Gja5* was used as an internal standard. ntc indicates no-template control. qRT-PCR showed that the transgene was most integrated into mouse number 13. (**C**) qRT-PCR analysis of NPGL precursor mRNA expression in the mediobasal hypothalamus of the WT and *Npgl* Tg mice. (**D**) Western blot analysis of mature NPGL in the mediobasal hypothalamus of the WT and *Npgl* Tg mice. Synthesized NPGL was used as the positive control (PC). (**E**) qRT-PCR analysis of NPGL precursor mRNA concentrations in different brain regions and intestines of *Npgl* Tg mice and in the MBH of WT mice. The NPGL precursor mRNA levels were quantified relative to the level of *Actb* mRNA. Each value represents the mean ± SEM (*n* = 2). *Npgl*, neurosecretory protein GL; CMV, cytomegalovirus; IRES, internal ribosome entry sites; *Gfp*, green fluorescent protein; *Gja5*, gap junction alpha 5; WT, wild-type; Tg, transgenic; MBH, mediobasal hypothalamus; *Actb*, β-actin.

Construction of the transgene is shown in Figure 1A. The ubiquitous cytomegalovirus (CMV) promoter was used to regulate the expression of *Npgl*. The transgene solution was microinjected into the pronuclei of fertilized eggs (ICR background), and the eggs were transferred into fallopian tubes of pseudo-pregnant female mice. As a result, 82 F0 mice were obtained. Genotyping revealed that the transgene was integrated into the genome of seven mice. The individual identification numbers were 7, 13, 17, 19, 22, 27, and 56 (Figure 1B). Quantitative analysis of integrated green fluorescent protein (*Gfp*) genes showed that integrated copies of the transgene were highest in the 13-line (Figure 1B). However, the amount of integrated transgene is not proportional to its expression level because the exogenous transgene is affected by the site of chromosomal integration [25,26]. Therefore, we analyzed the expression of *Npgl* mRNA in the mediobasal hypothalamus (MBH). The results show that the 13-line *Npgl* Tg mouse also had the highest level of *Npgl* mRNA expression among the five lines (Figure 1C). Furthermore, western blot analysis revealed that the mature NPGL protein was also overexpressed in the hypothalamus 13-line of *Npgl* Tg mice, and the levels were approximately 4.3 times higher than those in wild-type (WT) mice (Figure 1D). Thus, we mainly used the 13-line *Npgl* Tg mice for subsequent experiments. In addition, we used the 17-line *Npgl* Tg mice to confirm the phenotype as a different line.

Several studies have reported that the CMV promoter is prone to transcriptional silencing [27,28,29,30,31]. Therefore, the expression levels of *Npgl* mRNA in different tissues of *Npgl* Tg mice were examined using quantitative reverse transcriptase PCR (qRT-PCR). In *Npgl* Tg mice, *Npgl* mRNA was widely overexpressed in different regions of the brain, such as the cerebrum, diencephalon, mesencephalon, cerebellum, and MBH, whereas its expression was near the background level in the intestine (Figure 1E). In addition, the expression was not detected in the inguinal WAT, interscapular brown adipose tissue (BAT), or liver of *Npgl* Tg mice (data not shown). These results indicate that *Npgl* mRNA was overexpressed only in the central nervous system of *Npgl* Tg mice.

### 2.2. Phenotypic Analysis of Body Mass, Food Intake, and Peripheral Tissues

**Figure 2:**
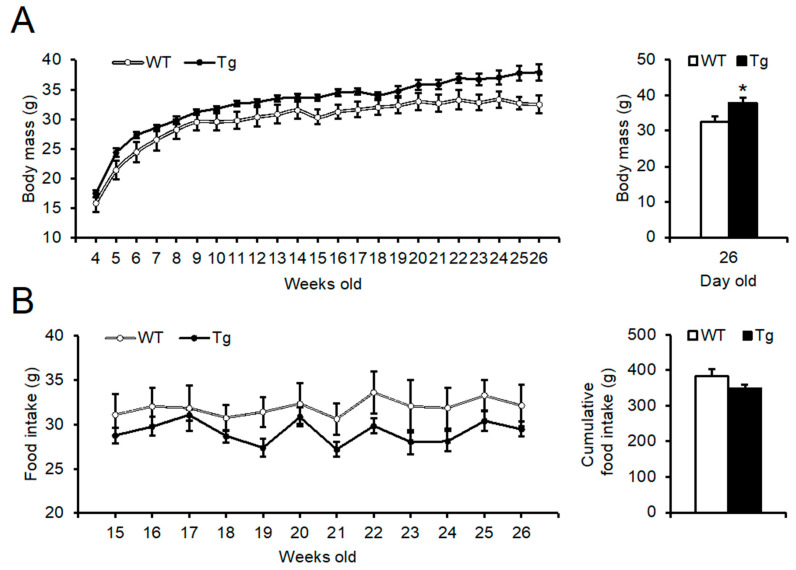

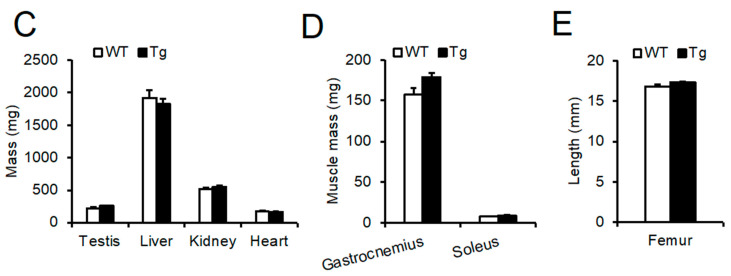
Phenotypic analysis of body mass, food intake, and peripheral tissues of the 13-line of *Npgl* Tg mice. (**A**) Weekly body mass changes (**left**) and body mass (**right**) at 26 weeks of age of WT and *Npgl* Tg mice. (**B**) Weekly food intake (**left**) and cumulative food intake (**right**) from 15 to 26 weeks of age. (**C**) The masses of the testis, liver, kidney, and heart. (**D**) The masses of the gastrocnemius and soleus muscles. (**E**) The length of the femur as an index of body length. Statistical analyses were performed using two-way repeated measures ANOVA followed by Bonferroni’s test (left of (**A**,**B**)) or Mann–Whitney U test (right of (**A**,**B**), and (**C**–**E**)). Each value represents the mean ± SEM (*n* = 5–8). Asterisks indicate statistically significant differences (* *p* < 0.05). *Npgl*, neurosecretory protein GL; WT, wild-type; Tg, transgenic.

To determine the effects of *Npgl* overexpression on the central nervous system of mice, we assessed body mass, food intake, and body composition. Although two-way repeated measures ANOVA revealed no significant effect of *Npgl* overexpression on body mass, that of *Npgl* Tg mice was higher than that of WT mice at 26 weeks of age (Figure 2A). WT and *Npgl* Tg mice showed similar food intake from 15 to 26 weeks of age (Figure 2B).

**Figure 3:**
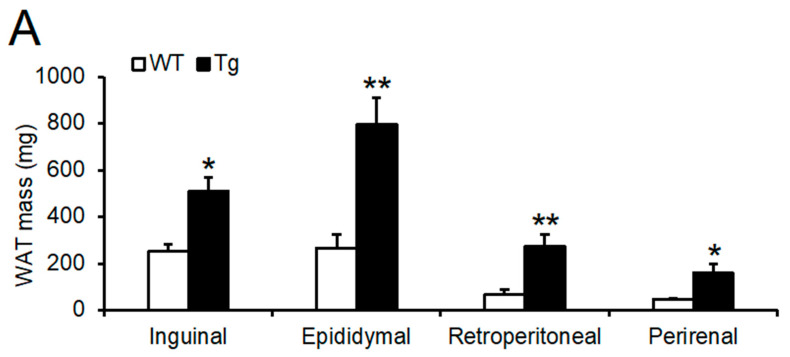

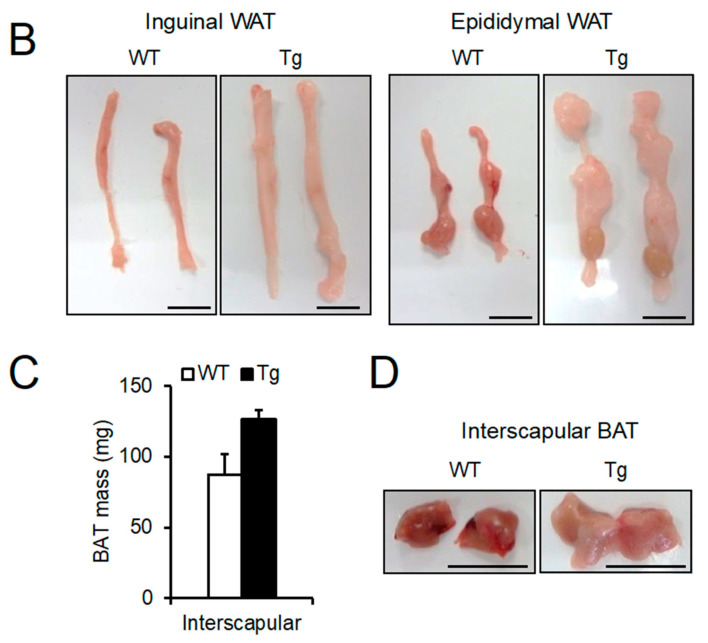
Phenotypic analysis of adipose tissues of the 13-line of *Npgl* Tg mice. (**A**) The masses of inguinal, epididymal, retroperitoneal, and perirenal WAT. (**B**) Representative photographs of inguinal and epididymal WAT. (**C**) The mass of interscapular BAT. (**D**) Representative photographs of interscapular BAT. Scale bars = 1 cm. All statistical analyses were performed using Mann–Whitney U test. Each value represents the mean ± SEM (*n* = 5–8). Asterisks indicate statistically significant differences (* *p* < 0.05, ** *p* < 0.01). *Npgl*, neurosecretory protein GL; WT, wild-type; Tg, transgenic; WAT, white adipose tissue; BAT, brown adipose tissue.

The masses of the testis, liver, kidney, and heart did not change (Figure 2C). The masses of the gastrocnemius and soleus muscles were also unaffected (Figure 2D). In addition, the femur length, an index of body length, did not change (Figure 2E). While there were no remarkable changes in the masses of internal organs, muscles, and the length of the femur, the masses of inguinal WAT, epididymal WAT, retroperitoneal WAT, and perirenal WAT were drastically increased in *Npgl* Tg mice (Figure 3A,B). The mass of interscapular BAT in *Npgl* Tg mice was not different from that in WT mice, but the BAT of *Npgl* Tg mice showed whitening (Figure 3C,D). *Npgl* Tg mice in the 17-line, in which the *Npgl* mRNA expression level was lower than that in the 13-line, also showed increases in the masses of WATs and BAT, similar to that in the 13-line (Figure S1).

To examine the effects of *Npgl* overexpression on the expression of several neuropeptides and hormones, we conducted qRT-PCR in the hypothalamus and pituitary gland in the 13-line of mice. The mRNA expression levels did not change significantly, except for follicle-stimulating hormone (*Fsh*) in the pituitary gland (Figure S2A,B).

### 2.3. Lipid Droplet Hypertrophy in Adipose Tissues

**Figure 4:**
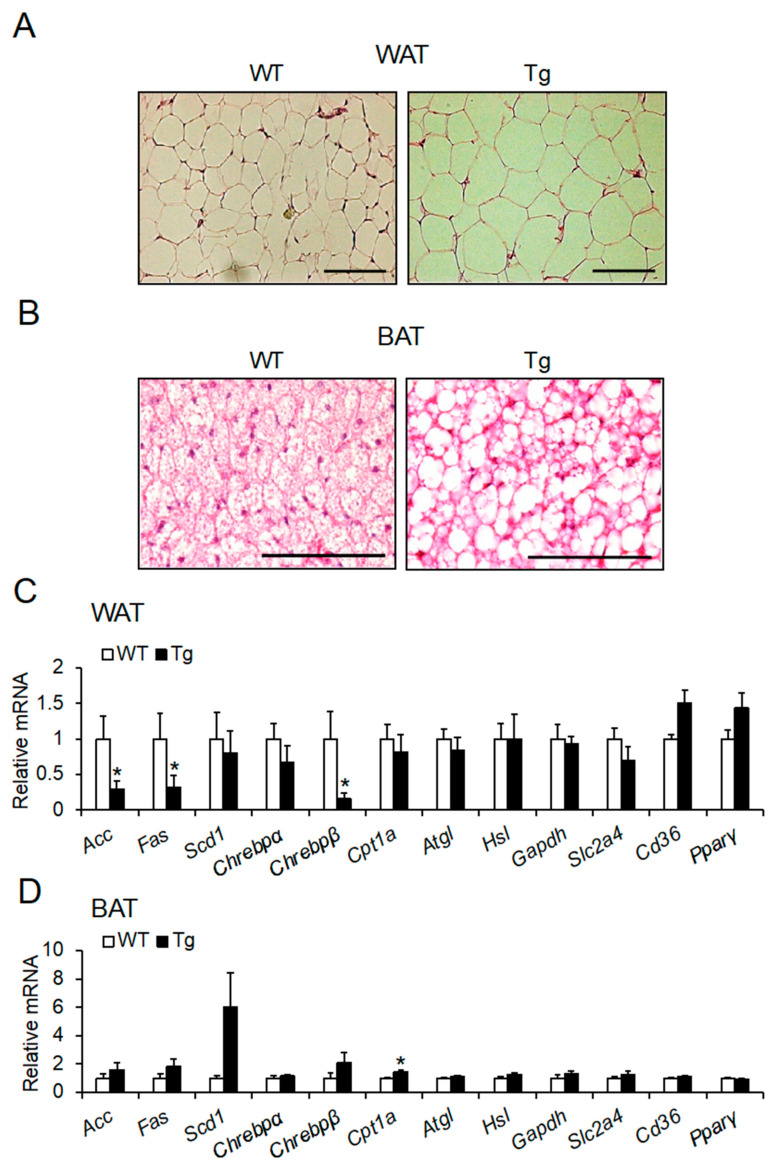
Morphological and mRNA expression analyses of adipose tissues of *Npgl* Tg mice. (**A**,**B**) Hematoxylin-eosin staining of epididymal WAT (**A**) and interscapular BAT (**B**) sections. Scale bars = 100 μm. (**C**,**D**) The mRNA expression levels of lipid metabolism-related genes in inguinal WAT (**C**) and interscapular BAT (**D**). All statistical analyses were performed using Mann–Whitney U test. Each value represents the mean ± SEM (*n* = 5–8). Asterisks indicate statistically significant differences (* *p* < 0.05). *Npgl*, neurosecretory protein GL; WT, wild-type; Tg, transgenic; WAT, white adipose tissue; BAT, brown adipose tissue.

We next performed histological analysis of WAT and BAT to determine whether the increase in WAT mass and whitening of BAT were associated with lipid droplet hypertrophy [32]. Histological analysis revealed that the droplets in WAT and BAT were hypertrophic (Figure 4A,B).

It is well known that serum leptin, an “anti-obesity” hormone secreted from WAT, increases during WAT increase. Therefore, we measured serum leptin concentration and observed that leptin levels were significantly elevated in *Npgl* Tg mice (Table 1). However, the serum levels of insulin, cholesterol, triglycerides, free fatty acids, and glucose did not change (Table 1).

### 2.4. mRNA Expression of Lipid Metabolic Factors in Adipose Tissues

As fat accumulation was accelerated in WAT and BAT, we expected that lipid metabolism in both adipose tissues would change in *Npgl* Tg mice. We measured the mRNA expression levels of genes involved in lipogenesis (acetyl-CoA carboxylase, *Acc*; fatty acid synthase, *Fas*; stearoyl-CoA desaturase 1, *Scd1*; and carbohydrate-responsive element-binding protein α and β, *Chrebp α and β*), lipolysis (carnitine palmitoyltransferase 1a, *Cpt1a*; adipose triglyceride lipase, *Atgl*; and hormone-sensitive lipase, *Hsl*), glycolysis (glyceraldehyde 3-phosphate dehydrogenase, *Gapdh*), glucose uptake (solute carrier family 2 member 4, *Slc2a4*), lipid uptake (cluster of differentiation 36, *Cd36*), and adipocyte differentiation (peroxisome proliferator-activated receptor γ, *Pparγ*). Although fat accumulation was observed in the inguinal WAT of *Npgl* Tg mice, the mRNA expression levels of *Acc*, *Fas*, and *Chrebpβ* were decreased (Figure 4C). In BAT, the mRNA expression of *Cpt1a* was upregulated in *Npgl* Tg mice (Figure 4D). Subsequently, to investigate the possibility that dysfunction in BAT thermogenesis mainly causes lipid droplet hypertrophy, we measured the mRNA expression levels of genes involved in thermogenesis in BAT, such as uncoupling protein 1 (*Ucp1*), type 2 iodothyronine deiodinase (*Dio2*), and peroxisome proliferator-activated receptor γ coactivator 1α (*Pgc1α*). However, no significant differences were observed (Figure S2C).

### 2.5. Histological Analysis and mRNA Expression of Lipid Metabolic Factors in the Liver

**Figure 5:**
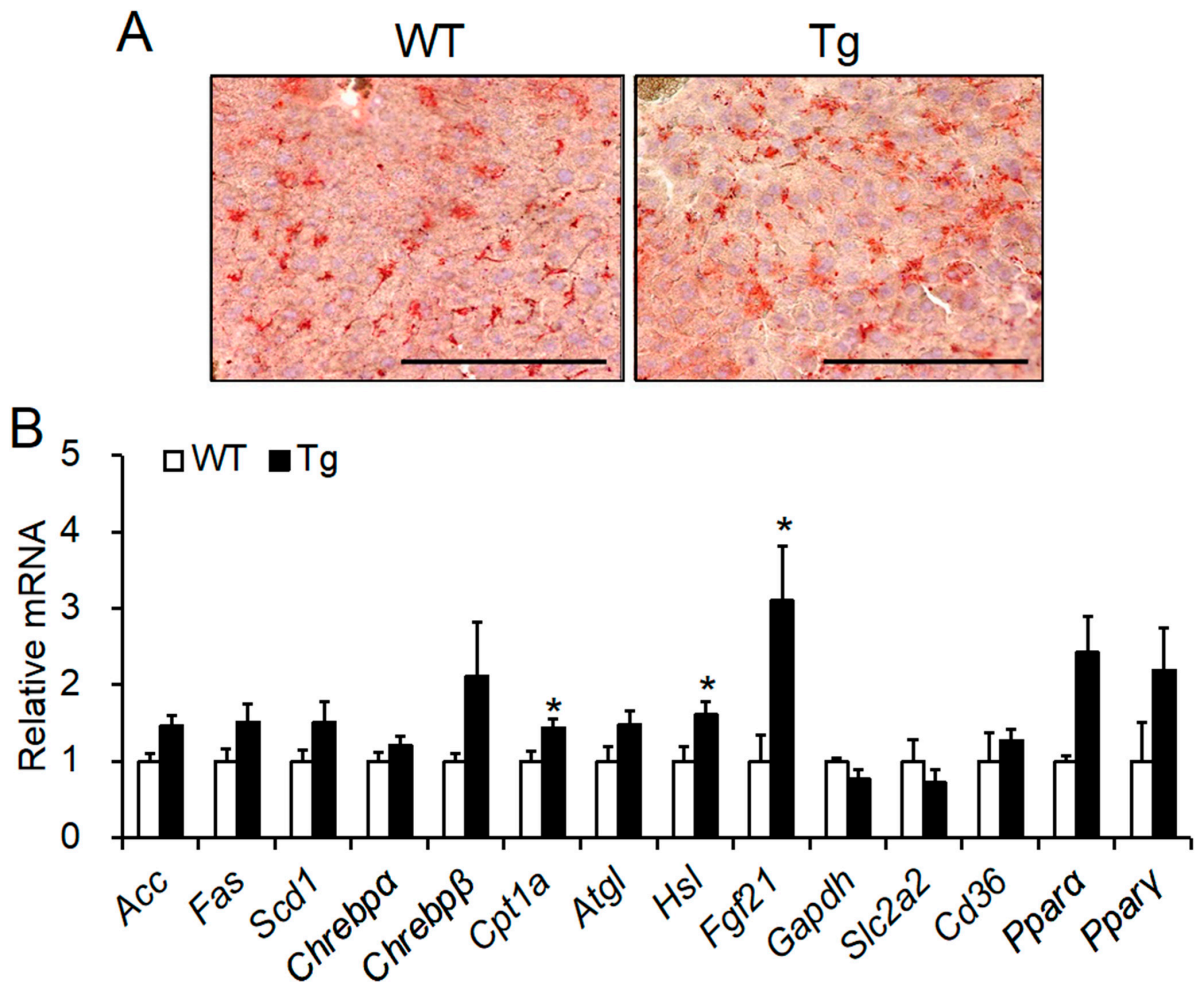
Morphological and mRNA expression analyses of the liver of *Npgl* Tg mice. (**A**) Oil red O staining of the liver sections. Red dots show lipid droplets. Scale bars = 100 μm. (**B**) The expression levels of mRNA for lipid metabolism-related genes in the liver. All statistical analyses were performed using Mann-Whitney U test. Each value represents the mean ± SEM (*n* = 5–8). Asterisks indicate statistically significant differences (* *p* < 0.05). *Npgl*, neurosecretory protein GL; WT, wild-type; Tg, transgenic.

Next, we evaluated whether *Npgl* overexpression led to hepatic steatosis. Hepatic fat content in *Npgl* Tg mice was not different from that in WT mice despite adiposity in WAT in *Npgl* Tg mice (Figure 5A). qRT-PCR analysis revealed that the expression levels of *Cpt1a*, *Hsl*, and fibroblast growth factor 21 (*Fgf21*) mRNA were significantly elevated in the livers of *Npgl* Tg mice (Figure 5B). These data suggest that the hepatic lipid oxidation system is accelerated in *Npgl* Tg mice.

## 3. Discussion

We have identified that a novel gene encoding a small secretory protein, NPGL, induces fat accumulation in chicks, rats, and obesity-prone C57BL/6J mice [21,23,24]. However, whether NPGL induces adiposity in different strains of mouse is poorly understood. We showed that *Npgl* Tg mice backcrossed onto ICR mice showed overexpression of *Npgl* in the central nervous system. *Npgl* Tg mice showed increased WAT mass without any changes in food intake. Although BAT mass did not change in *Npgl* Tg mice, lipid droplet hypertrophy was observed in BAT and WAT. In addition, fat accumulation was not induced in the liver because of accelerated hepatic lipolysis. Taken together, our data indicate that NPGL leads to obesity in several mouse strains.

The present study shows that *Npgl* Tg mice displayed fat accumulation without increasing food intake, upregulated mRNA expression levels of lipogenic factors in adipose tissues, and changes in blood parameters. We have previously reported that NPGL rapidly leads to obesity by reducing the energy expenditure in C57BL/6J mice [24]. The mRNA expression levels of lipogenic factors in WAT decrease with adiposity [33]. Therefore, our data suggest that NPGL initially downregulates energy consumption, resulting in fat accumulation. Recently, some reports have suggested that ICR mice are a more suitable model for metabolic research than C57BL/6J mice [34,35,36]. Daruma mice, which are genetically obese mouse models generated by the ICR strain, have shown adiposity similar to leptin-deficient *ob*/*ob* mice and leptin receptor-deficient *db/db* mice [37]. Moreover, ICR mice are susceptible to streptozotocin/nicotinamide-induced diabetes [38]. Additional studies to unravel the effects of NPGL on insulin sensitivity and glucose tolerance using the ICR strain will help to elucidate the biological mechanisms of metabolic syndromes in heterogeneous populations.

Although we have previously reported that NPGL has an orexigenic effect in rodents [21,24,39], WT and *Npgl* Tg mice showed similar food intake in this study. Several studies have shown differences in the effects of neuropeptides between pharmacological approaches and genetically modified animals. I.c.v. injection of NPY potently stimulates feeding behavior, although *Npy* knockout has little effect on food intake [40,41]. *Npy* transgenic rats also exhibit normal feeding behavior [42]. Furthermore, i.c.v. injection of AgRP accelerates feeding behavior, and *Agrp*-deficient mice display few changes in feeding behavior [43]. Recently, the analysis of chemogenetics or optogenetics has become a powerful tool for research on the biological actions of specific neurons [44,45,46,47]. Therefore, reversible manipulation of NPGL neurons using chemogenetics and/or optogenetics is required to evaluate the orexigenic effects of NPGL.

In *Npgl* Tg mice, we observed normotopic fat accumulation in the WAT and prevention of ectopic adiposity, such as the development of fatty liver. In addition, upregulation of *Cpt1a* and *Hsl* mRNA expression was detected in the livers of *Npgl* Tg mice. FGF21, an endocrine hormone that is mainly secreted from the liver, is regulated by ChREBP [48]. CPT1a and HSL are enzymes that catalyze β-oxidation. Hepatic *Cpt1a* expression levels are increased by *Fgf21* overexpression and reduced by *Fgf21* knockdown [49,50]. A recent study has demonstrated that ipragliflozin, an inhibitor of sodium-glucose cotransporter 2 (SGLT2), accelerates normotopic fat accumulation and inhibits liver steatosis [51]. These studies suggest that NPGL promotes normotopic fat accumulation in WAT and lipolysis in the liver via the inhibition of SGLT2. Further studies to elucidate the NPGL-SGLT2 system will provide new insights into normotopic fat accumulation and metabolic diseases.

One of the limitations in the present study may be the random overexpression of *Npgl* in mice. In this study, we addressed *Npgl* overexpression using CMV promoter. As CMV promoter allows gene expressions in ubiquitous regions, we observed the expression of *Npgl* in several brain regions including MBH and in other regions of *Npgl* Tg mice. On the other hand, we have previously reported that *Npgl* is highly expressed in the MBH, while it is slightly expressed in other brain regions [20]. Therefore, the region-specific expression will help to develop the research on the physiological function of NPGL. Moreover, to evaluate the effects of NPGL on lipid metabolism, we only conducted qRT-PCR in adipose tissues. Lipid metabolic factors are regulated by not only mRNA levels but also protein and/or phosphorylation levels. Our present study showed the few effects of lipid metabolic factors on mRNA levels, despite fat accumulation in adipose tissues. To clarify this discrepancy, further studies to evaluate the enzymatic activities and the protein abundance of lipid metabolic factor are required.

In conclusion, NPGL induces normotopic fat accumulation in WAT and prevents the development of fatty liver in ICR mice. This report strongly supports the notion that NPGL acts as a potent anabolic neuropeptide in several mouse strains. Considering that ICR strain mice are a more reasonable model for metabolic research than C57BL/6J mice [34,35,36], our findings open up new avenues for the comprehension of metabolic status and genetic polymorphisms. A recent study reported the importance of genotype-informed treatments owing to the association between obesity and genetic polymorphisms [52]. Future studies investigating the differences in the metabolic effects of NPGL using various mouse strains or species may contribute to the progress of obesity and metabolic syndrome in heterogeneous populations.

## 4. Materials and Methods

### 4.1. Animals

Male mice were kept at 25 ± 1 °C with a 12-h light/12-h dark cycle and provided *ad libitum* access to water and food (CE-2; CLEA Japan, Tokyo, Japan). Heterozygous transgenic mice were used in this study. 13-line *Npgl* Tg mice were housed individually from 4 weeks of age, and their body mass was measured every week. Food intake was measured from 15 to 26 weeks of age. 17-line *Npgl* Tg mice were divided into groups of five, and their body mass was measured weekly from 4 weeks of age. All animal experiments were performed in accordance with the Guide for the Care and Use of Laboratory Animals prepared by Hiroshima University (Higashi-Hiroshima, Japan), and the procedures were approved by the Institutional Animal Care and Use Committee of Hiroshima University (permit number: C14-1-2, 19 April 2016).

### 4.2. Generation of Transgenic Mouse Overexpressing Npgl

The transgenes used in this study are shown in Figure 1A. The 3.8 kb transgene was electrophoresed on 1% agarose gel, and the DNA band was cut out and purified with Wizard SV Gel and the PCR Clean-Up System (Promega, Madison, WI, USA). The transgene solution was microinjected into the host pronuclei of fertilized eggs from mice (ICR outbred strain), and the eggs were transferred into fallopian tubes of pseudo-pregnant female mice.

### 4.3. Selection of the Line of Npgl Tg Mice

The genotypes of all F0 mice were determined by genomic DNA analysis. Genomic DNA was isolated from tails using the PureLink Genomic DNA Mini Kit (Life Technologies, Carlsbad, CA, USA) and amplified with TaKaRa Ex Taq (TaKaRa Bio, Shiga, Japan) under the following conditions: 94 °C for 3 min, followed by 25 cycles of 94 °C for 30 s and 60 °C for 30 s. The amplified product was electrophoresed on 1% agarose gel. The gap junction alpha-5 protein (*Gja5*) gene was used as an endogenous control. Relative transgene integration was measured by qRT-PCR. qRT-PCR was performed with THUNDERBIRD SYBR qPCR Mix (TOYOBO, Osaka, Japan) using the following conditions: 95 °C for 20 s, followed by 40 cycles of 95 °C for 3 s, and 60 °C for 30 s. The *Gja5* gene was used as an endogenous control. The primers used in this study are listed in Table 2. The data were analyzed using the 2^−ΔΔCt^ method [53]. As a result, seven F0 transgenic mice were generated. These seven mice were mated with WT mice to produce the transgenic offspring. The transgene was inherited from the offspring of five F0 mice, and we successfully obtained five transgenic lines. To select a transgenic line for further analysis, we analyzed *Npgl* mRNA expression levels in the MBH using qRT-PCR.

### 4.4. Tissue Collection

The mice were immediately euthanized by decapitation. Subsequently, the MBH, pituitary gland, adipose tissues (WAT and interscapular BAT), internal organs (liver, kidney, heart, and testis), muscles (gastrocnemius and soleus), long bones (femur), and blood were collected from mice. We measured the masses of adipose tissues, internal organs, and the length of the bone. The MBH, pituitary gland, liver, inguinal WAT, and BAT were frozen in liquid nitrogen and stored at −80 °C for RNA processing. The liver, epididymal WAT, and BAT were fixed in 4% paraformaldehyde (PFA) solution and stored at 4 °C for histological analysis. Blood was centrifuged for 15 min at 800× *g* and 4 °C after incubation for 30 min at 25 °C, and the serum was stored at −40 °C.

### 4.5. qRT-PCR

Total RNA was isolated using TRIzol reagent (Life Technologies) for the MBH and liver, QIAzol reagent for the adipose tissues (QIAGEN, Venlo, The Netherlands), or the RNAqueous-Micro Scale RNA Isolation Kit for the pituitary gland (Life Technologies). First-strand cDNA was reverse transcribed using the ReverTra Ace kit (TOYOBO). qRT-PCR was performed with THUNDERBIRD SYBR qPCR Mix (TOYOBO) under the following conditions: 95 °C for 20 s, followed by 40 cycles of 95 °C for 3 s, and 60 °C for 30 s. Data were analyzed by the 2^−ΔΔCt^ method using β-actin (*Actb*) for the MBH, pituitary gland, liver, or ribosomal protein S18 (*Rps18*) for adipose tissues [53]. The primers used in this study are listed in Table 2.

### 4.6. Western Blot Analysis

To detect mature NPGL in the hypothalamus of WT and *Npgl* Tg mice, Western blot analysis was performed after SDS-PAGE following our previously published methods [21,24]. The hypothalamus of WT and *Npgl* Tg mice was boiled and homogenized. The homogenate was centrifuged at 15,000× *g* for 20 min at 4 °C. After extraction with dimethyl sulfoxide, the supernatant was subjected to reversed-phase HPLC using an octadecylsilane (ODS) column (TSK gel ODS-80Ts; 4.6 × 150 mm; Tosoh, Tokyo, Japan) with a linear gradient of 20−60% acetonitrile containing 0.1% trifluoroacetic acid at a flow rate of 0.5 mL/min. The fractions were evaporated and dissolved in SDS sample buffer. This solution was subjected to 15% SDS-PAGE. After transfer, the blot was detected with a rabbit antibody against NPGL (1:1000 dilution), horseradish peroxidase-labeled donkey anti-rabbit IgG (1:1000 dilution; GE Healthcare, Little Chalfont, England), and ECL Prime Western blotting detection reagent (GE Healthcare).

### 4.7. Hematoxylin and Eosin Staining

For paraffin embedding, epididymal WAT and interscapular BAT were fixed with 4% PFA solution for several days and delipidated with xylene and ethanol. Adipose tissues were embedded in paraffin and sectioned to a thickness of 7 µm using a microtome. The sections were air-dried and deparaffinized in a graded alcohol series. The nuclei and cytoplasm were stained with hematoxylin and eosin (5 min for each stain), and the sections were washed with tap water. After dehydration in a graded alcohol series and clearing with xylene, sections were mounted on slides and examined under a microscope.

### 4.8. Oil Red O Staining

To detect fat accumulation in the liver, hepatic tissue was fixed in 4% paraformaldehyde and sliced into 10-µm-thick sections. Sections were air-dried, rinsed with 60% isopropanol, stained with Oil Red O solution for 15 min at 37 °C, and rinsed with 60% isopropanol. The nuclei were counterstained with hematoxylin for 5 min, and the sections were washed with tap water. Coverslips were applied using an aqueous mounting medium, and microscopic examination was performed using a microscope.

### 4.9. Serum Biochemical Analysis

Glucose content was measured using a glucose content monitor (Arkray, Kyoto, Japan). The NEFA C-Test Wako (Wako Pure Chemical Industries, Osaka, Japan) was used to measure free fatty acid levels. The Triglyceride E-Test Wako (Wako Pure Chemical Industries) was used to measure triglyceride levels, the Cholesterol E-Test Wako (Wako Pure Chemical Industries) for cholesterol content, the Leptin ELISA kit (Morinaga Institute of Biological Science, Kanagawa, Japan) for leptin, and the LBIS Insulin Mouse T ELISA Kit (Shibayagi, Gunma, Japan) for insulin.

### 4.10. Statistical Analysis

Mann–Whitney U test was performed to assess the 2-group differences between WT and *Npgl* Tg mice. To assess the main effects of groups (WT and Tg) and time, two-way ANOVA with repeated measures followed by Bonferroni’s test for multiple comparisons was conducted. Differences with *p* values < 0.05 were considered statistically significant. All results are presented as the mean ± standard error of the mean (SEM).

## Figures and Tables

**Table 1:** Serum biochemical parameters. All statistical analyses were performed using Mann-Whitney U test. Each value represents the mean ± SEM (*n* = 5–7). Asterisk indicates a statistically significant difference (* *p* < 0.05).

	WT	Tg
Leptin (ng/mL)	0.676 ± 0.134	1.96 ± 0.438 *
Insulin (ng/mL)	1.06 ± 0.270	0.973 ± 0.149
Cholesterol (mg/dL)	123 ± 7.19	113 ± 4.64
Triglycerides (mg/dL)	162 ± 12.27	139 ± 16.0
Free fatty acids (mEq/L)	1.70 ± 0.256	1.84 ± 0.182
Glucose (mg/dL)	129 ± 6.11	136 ± 7.88

**Table 2:** Sequences of oligonucleotide primers for qRT-PCR.

Gene	Sense Primer (5′ to 3′)	Antisense Primer (5′ to 3′)
*Gfp*	ACCACTACCTGAGCACCCAGTC	GTCCATGCCGAGAGTGATCC
*Gja5*	ACCATGGAGGTGGCCTTCA	CATGCAGGGTATCCAGGAAGA
*Npgl*	GGAACCATGGCTTAGGAAGG	TCTAAGGAGCTGAGAATATGCA
*Acc*	TCCGCACTGACTGTAACCACAT	TGCTCCGCACAGATTCTTCA
*Fas*	AGGGGTCGACCTGGTCCTCA	GCCATGCCCAGAGGGTGGTT
*Scd1*	CTGTACGGGATCATACTGGTTC	GCCGTGCCTTGTAAGTTCTG
*Chrebpα*	CGACACTCACCCACCTCTTC	TTGTTCAGCCGGATCTTGTC
*Chrebpβ*	TCTGCAGATCGCGTGGAG	CTTGTCCCGGCATAGCAAC
*Cpt1a*	CCTGGGCATGATTGCAAAG	GGACGCCACTCACGATGTT
*Atgl*	AACACCAGCATCCAGTTCAA	GGTTCAGTAGGCCATTCCTC
*Hsl*	GCTGGGCTGTCAAGCACTGT	GTAACTGGGTAGGCTGCCAT
*Gapdh*	AAGGTCATCCCAGAGCTGAA	CTGCTTCACCACCTTCTTGA
*Slc2a4*	GTAACTTCATTGTCGGCATGG	AGCTGAGATCTGGTCAAACG
*Cd36*	TCCTCTGACATTTGCAGGTCTATC	AAAGGCATTGGCTGGAAGAA
*Pparγ*	GCCCTTTGGTGACTTTATGGA	GCAGCAGGTTGTCTTGGATG
*Fgf21*	CCTCTAGGTTTCTTTGCCAACAG	AAGCTGCAGGCCTCAGGAT
*Slc2a2*	GGCTAATTTCAGGACTGGTT	TTTCTTTGCCCTGACTTCCT
*Pparα*	TCGAATATGTGGGGACAAGG	GACAGGCACTTGTGAAAACG
*Npy*	TATCTCTGCTCGTGTGTTTG	GATTGATGTAGTGTCGCAGA
*Agrp*	TGTTCCCAGAGTTCCCAGGTC	GCATTGAAGAAGCGGCAGTAGCAC
*Pomc*	AGCTGCCTTTCCGCGACA	ATCTATGGAGGTCTGAAGCA
*Ghrh*	TGCCATCTTCACCACCAAC	TCATCTGCTTGTCCTCTGTCC
*Sst*	GAGGACCTGCGACTAGACTGAC	CAGCAGCTCTGCCAAGAAGTA
*Trh*	TCGTGCTAACTGGTATCCCC	CCCAAATCTCCCCTCTCTTC
*Tsh*	CACCATCTGTGCTGGGTATTG	CATCCTGGTATTTCCACCGTTC
*Lh*	TGGCCGCAGAGAATGAGTTC	ACTCGGACCATGCTAGGACA
*Fsh*	GGAGAGCAATCTGCTGCCAT	GCCGAGCTGGGTCCTTATAC
*Prl*	GGCTACACCTGAAGACAAGGAACAA	TGTTCCTCAATCTCTTTGGCTCTTG
*Gh*	GGAGGCTAGTGCTTTTCCCG	AGGCACGCTCGAACTCTTTG
*Ucp1*	CAAAAACAGAAGGATTGCCGAAA	TCTTGGACTGAGTCGTAGAGG
*Dio2*	CCACCTTCTTGACTTTGCCA	GGTGAGCCTCATCAATGTATAC
*Pgc1α*	GCAACATGCTCAAGCCAAAC	TGCAGTTCCAGAGAGTTCCA
*Actb*	GGCACCACACCTTATACAAT	AGGTCTCAAACATGATCTGG
*Rps18*	CCTGAGAAGTTCCAGCACAT	TTCTCCAGCCCTCTTGGTG

## Data Availability

The raw data supporting the findings of this manuscript will be made available by the corresponding author, K.U., to any qualified researchers upon reasonable request.

## Supplementary Materials

The following supporting information can be downloaded at: https://www.mdpi.com/article/10.3390/ijms23126488/s1.

## Abbreviations

ACC	Acetyl-CoA carboxylase
ACTB	β-Actin
AgRP	Agouti-related protein
ATGL	Adipose triglyceride lipase
α-MSH	α-melanocyte-stimulating hormone
BAT	Brown adipose tissue
CART	Cocaine- and amphetamine-regulated transcript
CD36	Cluster of differentiation 36
ChREBPα	Carbohydrate-responsive element-binding protein α
ChREBPβ	Carbohydrate-responsive element-binding protein β
CMV	Cytomegalovirus
COVID-19	Coronavirus disease 2019
CPT1a	Carnitine palmitoyltransferase 1a
CRH	Corticotropin-releasing hormone
DIO	Diet-induced obesity
DIO2	Type 2 iodothyronine deiodinase
FAS	Fatty acid synthase
FGF21	Fibroblast growth factor 21
FSH	Follicle stimulating hormone
GAPDH	Glyceraldehyde 3-phosphate dehydrogenase
GFP	Green fluorescent protein
GH	Growth hormone
GHRH	Growth hormone releasing hormone
GJA5	Gap junction alpha-5 protein
HFD	High-fat diet
HSL	Hormone-sensitive lipase
i.c.v.	Intracerebroventricular
IRES	Internal ribosome entry sites
LH	Luteinizing hormone
MBH	Mediobasal hypothalamus
MCH	Melanin-concentrating hormone
NPGL	Neurosecretory protein GL
NPY	Neuropeptide Y
ODS	Octadecylsilane
PFA	Paraformaldehyde
PC	Positive control
PGC1α	Peroxisome proliferator-activated receptor-γ coactivator 1α
POMC	Proopiomelanocortin
PPARα	Peroxisome proliferator-activated receptor α
PPARγ	Peroxisome proliferator-activated receptor γ
PRL	Prolactin
qRT-PCR	Quantitative reverse transcriptase PCR
RPS18	Ribosomal protein S18
SCD1	Stearoyl-CoA desaturase 1
SGLT2	Sodium-glucose cotransporter 2
SLC2A2	Glucose transporter 2
SLC2A4	Glucose transporter 4
SST	Somatostatin
Tg	Transgenic
TRH	Thyrotropin-releasing hormone
TSH	Thyroid stimulating hormone
UCP1	Uncoupling protein 1
WAT	White adipose tissue
WT	Wild-type
