# Supplementary material for: Neurosecretory Protein GL Promotes Normotopic Fat Accumulation in Male ICR Mice
Source: Int J Mol Sci. 2022 Jun 10;23(12):6488. doi: 10.3390/ijms23126488 (PMC9223635; doi:10.3390/ijms23126488)
Supplement: Supplementary file 1 [file ijms-23-06488-s001.zip › ijms-1737105-supplementary.pdf]

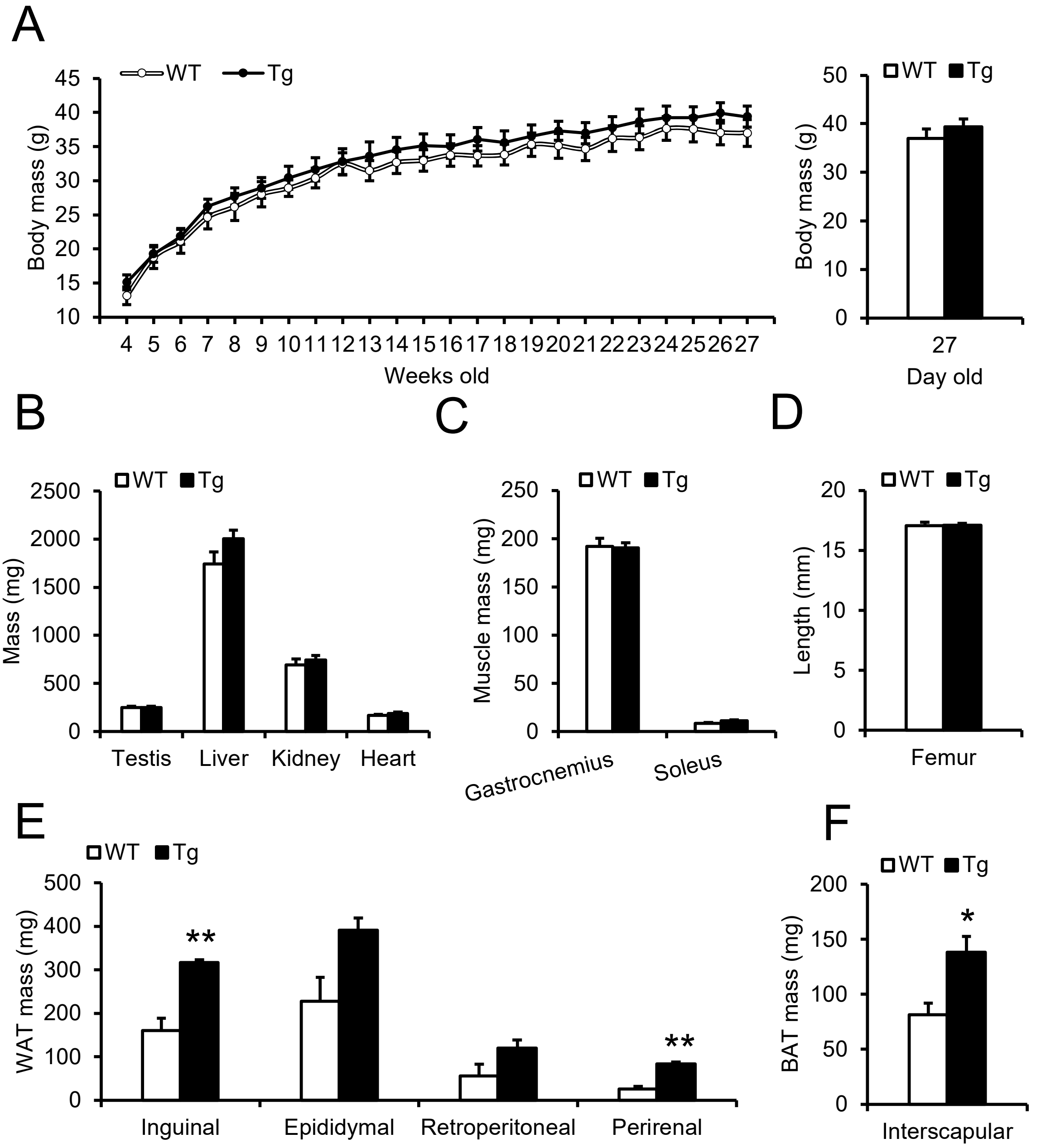

Supplementary Figure S1.

Phenotypic analysis of body mass and peripheral tissues of the 17-line of *Npgl* Tg mice. (**A**) Weekly body mass changes (left) and body mass (right) at 27 weeks of age of WT and *Npgl* Tg mice. (**B**) The masses of the testis, liver, kidney, and heart. (**C**) The masses of gastrocnemius and soleus muscles. (**D**) The length of the femur as an index of body length. (**E**) The masses of inguinal, epididymal, retroperitoneal, and perirenal WAT. (**F**) The mass of interscapular BAT. All statistical analyses were performed using Mann-Whitney U test. Each value represents the mean ± SEM (n = 5). Asterisks indicate statistically significant differences (*$P < 0.05$, **$P < 0.01$). *Npgl*, neurosecretory protein GL; WT, wild-type; Tg, transgenic; WAT, white adipose tissue; BAT, brown adipose tissue.

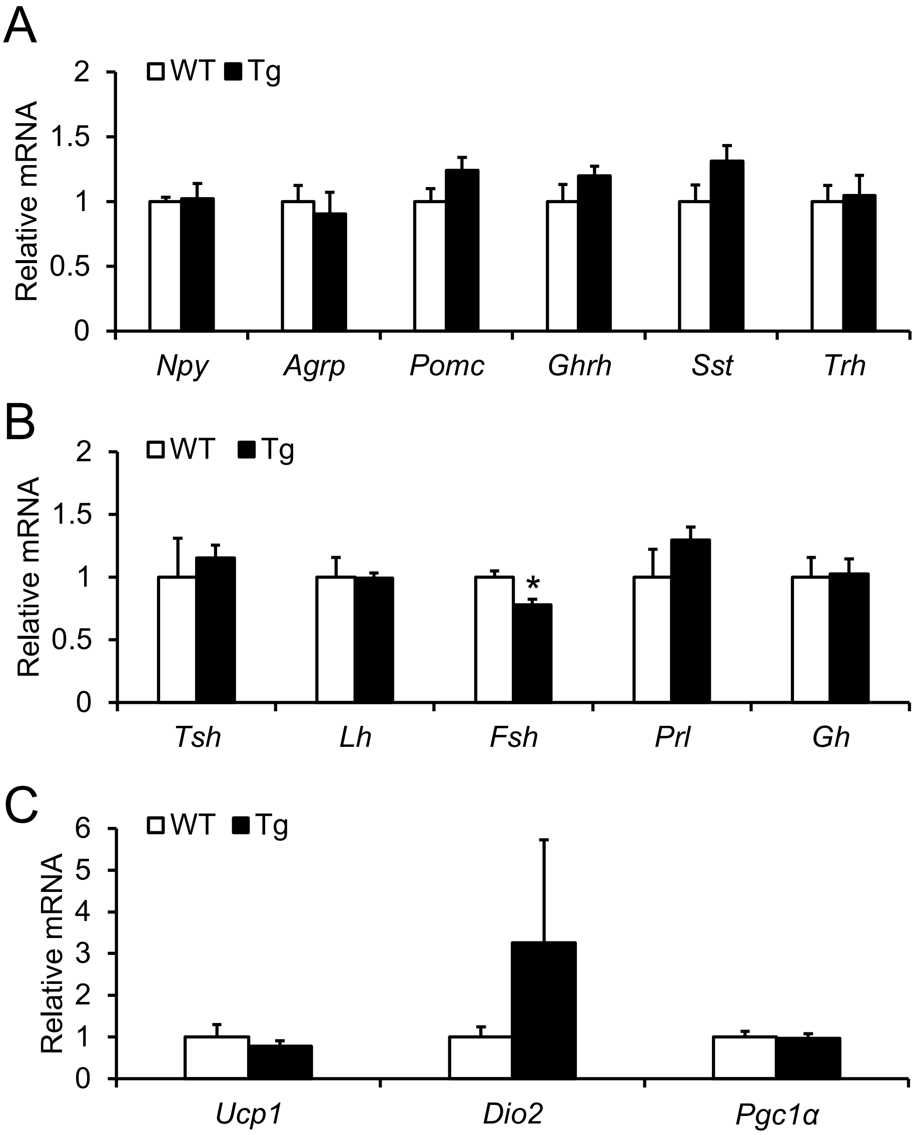

Supplementary Figure S2.

mRNA expression analysis of adipose tissues of *Npgl* Tg mice. (**A**) The mRNA expression levels of *Npy, Agrp, Pomc, Ghrh, Sst,* and *Trh* in the mediobasal hypothalamus. (**B**) The mRNA expression levels of *Tsh, Lh, Fsh, Prl* and *Gh* in the pituitary gland. (**C**) The mRNA expression levels of *Ucp1, Dio2,* and *Pgc1α* in interscapular BAT. All statistical analyses were performed using Mann-Whitney U test. Each value represents the mean ± SEM (n = 5–8). Asterisk indicates a statistically significant difference (*$P < 0.05$). *Npgl*, neurosecretory protein GL; WT, wild-type; Tg, transgenic; *Npy*, neuropeptide Y; *Agrp*, agouti-related peptide; *Pomc*, proopiomelanocortin; *Ghrh*, growth hormone-releasing hormone; *Sst*, somatostatin; *Trh*, thyrotropin-releasing hormone; *Tsh*, thyroid-stimulating hormone; *Lh*, luteinizing hormone; *Fsh*, follicle-stimulating hormone; *Prl*, prolactin; *Gh*, growth hormone; BAT, brown adipose tissue; *Ucp1*, uncoupling protein 1; *Dio2*, type 2 iodothyronine deiodinase; *Pgc1α*, peroxisome proliferator-activated receptor-γ coactivator 1α.
